# A short history of thrombectomy – Procedure and success analysis of different endovascular stroke treatment techniques

**DOI:** 10.1177/1591019920961883

**Published:** 2020-11-09

**Authors:** B Friedrich, T Boeckh-Behrens, V Krüssmann, S Mönch, J Kirschke, K Kreiser, M Berndt, M Lehm, S Wunderlich, C Zimmer, J Kaesmacher, C Maegerlein

**Affiliations:** 1 Department of Diagnostic and Interventional Neuroradiology, Klinikum rechts der Isar, Technical University Munich, Munich, Germany; 2 Department of Radiology, University Hospital, LMU Munich, Germany; 3 Department of Radiology, München Klinik, Munich, Germany; 4 Department of Neurology, Klinikum rechts der Isar, Technical University Munich, Munich, Germany; 5 University Institute of Diagnostic and Interventional Neuroradiology, University Hospital Bern, Inselspital, University of Bern, Bern, Switzerland; 6 Department of Neurology, University Hospital Bern, Inselspital, University of Bern, Bern, Switzerland; 7 University Institute of Diagnostic, Interventional and Pediatric Radiology, University Hospital Bern, University of Bern, Bern, Switzerland

**Keywords:** Ischemic stroke, mechanical thrombectomy, thrombectomy techniques, historical development

## Abstract

**Background:**

The historical development of interventional stroke treatment shows a wide variation of different techniques and materials used. Thus, the question of the present work is whether the technical and procedural differences of thrombectomy techniques lead to different technical and clinical results.

**Methods and results:**

Analysis of a mixed retrospective/prospective database of all endovascular treated patients with an occlusion of the Carotid-T or M1 segment of the MCA at a single comprehensive stroke center since 2008. Patients were classified regarding the technical approach used. Six hundred sixty-eight patients were available for the final analysis. Reperfusion rates ranged between 56% and 100% depending on the technical approach. The use of balloon guide catheters and most recently the establishment of combination techniques using balloon guide catheters, aspiration catheters and stent retrievers have shown a further significant increase in the rates of successful recanalization, full recanalization and first-pass recanalization. Additionally, the technical development of interventional techniques has led to a subsequent drop in complications, embolization into previously unaffected territories in particular.

**Conclusion:**

Technical success of MT has improved substantially over the past decade owing to improved materials and procedural innovations. Combination techniques including flow modulation have emerged to be the most effective approach and should be considered as a standard of care.

**Level of evidence:** Level 3, retrospective study.

## Introduction

Although the first attempts at intra-arterial (i.a.) therapy of emergent large vessel occlusions (LVO) were already being conducted in the early 1980s, based upon attempts to dissolve the thrombus by the application of intraarterial drugs, it took more than 20 years to develop the first promising approaches to using mechanical clot retraction techniques^1–3^ and implementing first generation mechanical thrombectomy (MT) devices like the Merci device (Concentric Medical, San Francisco, CA, USA),^4^ the Phenox Clot Retriever (Phenox GmbH, Bochum, Germany),^5,6^ or the Penumbra Separator (Penumbra, Alameda, CA, USA),^7^ partially combined with i.a. thrombolysis as individual treatments. Although hereby technical success could be further increased, evidence was still lacking that such approaches could result in better clinical outcomes.^8–10^

Finally, after the implementation of so-called stent retrievers as thrombectomy devices, five randomized controlled trials in 2015 demonstrated impressively the effectivity of mechanical thrombectomy with thrombolysis in comparison to thrombolysis alone,^11–15^ leading to adaptions in stroke guidelines worldwide with stent retrievers as thrombectomy devices recommended as a first line technique in LVOs.

Therefore, at this point, the technical evolution seemed to have reached its preliminary peak and the development of further improvements was generally considered as probably of minor relevance.

However, this perception seems to have been premature, as several further technical and procedural changes, adaptions, combinations or new developments have shown partially substantial improvements in technical and clinical success of the thrombectomy procedure, e.g. sole aspiration maneuvers (ADAPT, A Direct Aspiration First Pass Technique),^16–19^ the use of balloon guiding catheters (BGC) instead of normal guiding catheters or long sheaths,^20–22^ the combined use of stent retrievers, distal access catheters and BGC and/or the withdrawal of the stent retriever only partially retracted into the distal access catheter compared with primary complete retractions of the stent retriever into the distal access catheter.^23,24^

As endovascular stroke treatment has been performed frequently at the author’s center since 2008 we have gone through the above-mentioned evolutions. Thus, the question of the present work is whether and to what extent the technical and procedural differences of thrombectomy techniques may lead to different technical and clinical results.

## Materials and methods

Analysis of a mixed retrospective/prospective database of all endovascular-treated patients at a single comprehensive stroke center since 2008 was performed. All patients who had undergone endovascular treatment of LVO of the carotid-T or the M1 segment of the middle cerebral artery (MCA) were identified. Subsequently, these patients were classified by two experienced neurointerventionalists (CM, BF) in consensus regarding the technical approach. The following categories of intervention techniques were differentiated:

1. Old Device: MT with exclusive application of first generation devices: MERCI, Phenox Clot Retriever, Penumbra Separator, and intra-arterial rtPA.
2. Guiding Catheter + Stent Retriever (no distal access or aspiration catheter):

The following guiding catheters were used: NeuronMAX 088 (Penumbra), and VISTA BRITE TIP (Cordis, Milpitas, CA, USA). The following stent retrievers were used: pREset (Phenox, Bochum, Germany), Solitaire (EV3, Irvine, CA, USA), and TREVO/TREVO XP (Stryker, Kalamazoo, MI, USA).

3. Guiding Catheter + Distal Access Catheter + Stent Retriever:

The following catheters were defined and used as distal access catheters: DAC (Concentric), Navien Intracranial Support Catheter (Covidien, Dublin, Ireland), NeuroBridgeIntermediate Catheter (Acandis GmbH, Pforzheim, Germany), and ReFlex (Reverse Medical Corporation, Irvine, CA, USA).

4. Guiding Catheter + Aspiration Catheter + Stent Retriever:

The following catheters were defined and used as aspiration catheters: 5MAX ACE, ACE 64, ACE 68 (Penumbra)/SOFIA, SOFIA Plus (MicroVention, CA, USA), and Catalyst 6 (Stryker)

5. Guiding Catheter + Aspiration Catheter without stent retriever application (ADAPT).
6. BGC + Stent Retriever:

The following BGCs were used: Cello (Medtronic, Dublin, Ireland), Flowgate/Flowgate2 (Stryker), and Merci (Stryker).

7.   PROTECT: PRoximal balloon Occlusion TogEther with direCt Thrombus aspiration during stent retriever thrombectomy^25^: BGC + Aspiration Catheter + Stent Retriever (complete retrieval of the stent retriever into the aspiration catheter).
8. PROTECT^PLUS^ (BADDASS): BGC + Aspiration Catheter + Stent Retriever (only partial retrieval of the stent retriever into the aspiration catheter and withdrawal of both as a unit into the BGC).^24,26^

Patients in whom either the technique could not be clearly identified or in whom mixed techniques were performed (change of technique within one intervention) were excluded. All patients for whom permanent stent implantation (extra- or intracranial) was necessary were also excluded.

The modified thrombolysis in cerebral infarction (mTICI)^27^ score was determined by the two neurointerventionalists mentioned above. Technical success was defined as mTICI 2 b/3. The number of maneuvers required in each case to achieve the final mTICI result was determined. Further, all included cases were analyzed in consensus on the occurrence of embolization to new territories (ENT). The procedure times, in particular the groin puncture and reperfusion times, were taken from the existing database. If no successful recanalization was achieved (mTICI <2 b), the control series after the last maneuver was used as the time endpoint.

Based on the retrospective analysis of the present study, written consent was waived by the local ethics committee.

### Statistical analysis

The statistical analysis of the available data was carried out using SPSS 25 (IBM, USA). To investigate whether there is a correlation between the technique used and the procedural parameters or time, a Spearman ρ correlation was performed. Differences between the groups were tested using the Kruskal-Wallis test. Statistical significance was assumed at p < 0.05. All data are presented as median (IQR) unless otherwise noted.

## Results

Between 01/01/2008 and 06/01/2018 a total of 786 patients were treated endovascularly in our center for an isolated M1 occlusion or an occlusion of the carotid-T. Of these, 30 patients had to be excluded from the analysis as the technical approach could not be clearly identified. A further 88 patients had to be excluded as mixed techniques were used in these patients (e.g. patients in whom repeated use of the ADAPT technique did not lead to success and subsequently a stent retriever-based procedure was performed or old devices and stent retrievers were used in the same procedure). Thus, 668 patients were available for the final analysis. In terms of patient characteristics, there were some substantial differences between the different treatment groups, particularly in terms of age and gender. However, there were no differences in the distribution of vascular occlusions or the initial NIHSS between the groups (Table 1). There was also no significant difference in the rate of systemic thrombolysis therapy between the treatment groups. However, the individual interventional techniques showed significant differences in procedural data and technical success (Table 1).

**Table 1.** Patient characteristics and procedure parameters.

N = 668	Old device (N = 34)	GC + Stentretriever (N = 12)	GC + DA + Stentretriever (N = 188)	GC + Aspirationcatheter + Stentretriever (N = 245)	BGC + Stentretriever (N = 10)	PROTECT (N = 87)	PROTECT+ (N = 33)	GC + Aspiration (ADAPT) (N = 59)	p-Value
Period used	2008–2009	2009–2012	2009–2013	2013–2018	2011–2017	2016–2017	2017–2018	2014–2018	
Average experience of operators (years)	2 (1 – 2)	4 (1–3)	4 (1–3)	5 (1–4)	5 (1–4)	5 (1–4)	6 (1–5)	5 (1–4)	0,154
Patient Age (years)	65 +/− 15	63 +/− 18	71 +/− 14	75 +/− 13	66 +/− 21	74 +/− 12	72 +/− 13	73 +/− 14	0.003
Patient Sex (female)	61.8 %	50 %	51.6 %	56.7 %	40 %	47.4 %	42.5	52.5 %	<0.001
Occlusion									0.243
M1	73.5 %	83.3 %	75.5 %	78 %	50 %	71.2 %	60.6 %	76.3 %	
Carotid-T	26.5 %	16.7 %	24.5 %	22 %	50 %	28.8 %	39.4 %	23.7 %	
Onset to groin	312 +/− 230 min	279 +/− 85 min	251 +/− 83 min	266 +/− 91 min	245 +/ 110 min	241 +/− 81 min	301 +/ 165 min	212 +/− 109 min	0.791
NIHSS (IQR)	15 (11–18)	15 (11–19)	16 (13–18)	15 (12–19)	16 (15–18)	17 (12–21)	15 (14–18)	17 (13–18)	0.896
i.v. rtPA	70.6 %	58.3 %	69.5 %	69.5 %	60 %	66.7 %	67.2 %	55.3%	0.732
AF	43%	36%	45%	42%	31%	46%	40%	35%	0.601
No. of maneuvers	3 (1–14)	2 (1–9)	3 (1–13)	2 (1–13)	1 (1–6)	2 (1–10)	1 (1–12)	1 (1–10)	<0.001
Groin to reperfusion	90 +/− 44	74 +/− 65	81 -/+ 49	68 +/− 49	56 +/− 59	42 +/− 32	23 +/− 16	42 +/− 43	<0.001
Successful recanalization	55.9 %	100 %	72.9 %	82 %	90 %	90.8 %	93.9 %	83.1 %	<0.001
TICI 3	11.8 %	16.7 %	20.7 %	36.7 %	40 %	57.5 %	75.8 %	49.2 %	<0.001
First pass TICI 3	0 %	0 %	3.7 %	10.7 %	20 %	25.3 %	51.5 %	40.7 %	<0.001
ENT	14.7 %	16.7%	9 %	5.7%	10 %	3.4 %	0 %	4.9 %	0.017

The successful reperfusion rates ranged between 56% and 100% depending on the technical approach that was chosen for MT.

**Figure 1.**
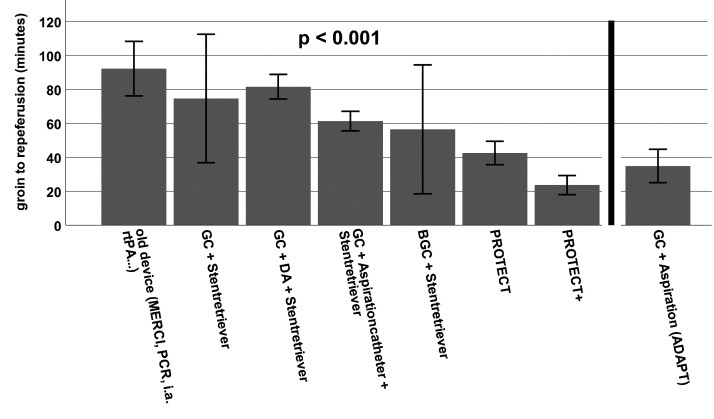
Procedure time of the different interventional stroke treatment techniques. Data are shown as mean. Whiskers indicate SEM.

**Figure 2.**
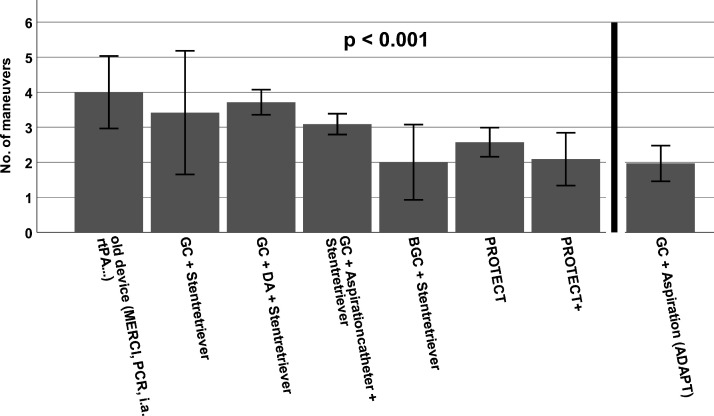
Number of maneuvers needed to achieve final angiographic result by the different endovascular techniques. Data are shown as mean. Whiskers indicate SEM.

**Figure 3.**
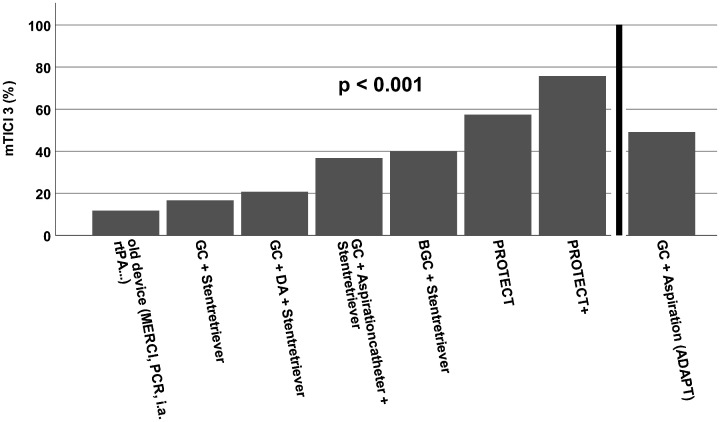
Rate of mTICI3 (complete) reperfusions that could be achieved by the different techniques analyzed here.

The use of BGCs showed an increase in the success rate to values above 90% (Tables 1 and 2). Similar changes were shown in the number of maneuvers and the procedure time. While recanalizations with old devices lasted 90 minutes and required three maneuvers, further technical developments led to a significant reduction of median maneuvers and procedure times to achieve the final angiographic result (Figure 1). In contrast to the old devices, PROTECT^PLUS^ for example required a median of one maneuver and achieved the result in 23 minutes (Figures 1 and 2). Regarding the rate of mTICI 3 reperfusions and first-pass mTICI 3 reperfusions we also found significant differences between the respective techniques (Figure 3). The rate of emboli in previously unaffected vascular territories (ENT) is another established safety parameter. Here every technical development showed a further reduction in the ENT rate; ENTs occurred in almost 15% of the cases of recanalizations using old devices, while first the use of stent retrievers and finally the combined use of additional BGCs reduced this rate to 0% with the development of PROTECT^PLUS^

**Table 2.** Procedure parameters: non-BGC: n = 504 (except Old Decices) versus BGC: n = 130.

	non-BGC	BGC	p-Value
No. of maneuvers	2 (1–4)	2 (1–3)	0.001
Groin to reperfusion (min)	66 +/− 48	39 +/− 32	<0.001
Successful recanalization	79.2%	91.5%	<0.001
TICI 3	31.7%	60.8%	<0.001
First pass TICI 3	11.3%	31.5%	<0.001
ENT	8.8%	3.1%	0.03

Comparing the procedures using BGC with those using non-BGC there was a significant advantage for the BGC group concerning all procedural parameters (Table 2, Figure 5).

## Discussion

Over the past decade, endovascular techniques have gone through a notable development, which was decisively boosted by technical developments like better catheters, new thrombectomy devices and other helpful neurointerventional tools. Interestingly, there are no guidelines so far that recommend the kind of material that should be used and in what way it should be used exactly for MT, except for the recommendation to use stent retrievers.^28^ For this reason, there are still great differences concerning technical approaches between different institutions, which can in part be explained by the lack of class 1 evidence regarding the comparison of different technical approaches but might also be due to department-specific preferences and different financial resources.

The recommendation to use stent retrievers is very well reproducible from our present data as all procedure parameters were significantly worse when using first generation devices like the merci, the phenox clot retriever, or i.a. rtPA (Table 1). This is in-line with three trials that showed no benefit of endovascular therapy regimes over i.v. rtPA alone when using such first-generation devices.^8–10^ One absolute exception is the pure aspiration technique (ADAPT). The comparatively good results of this apparently straightforward technique at first sight, are biased by the fact that the conversion rates are high when starting with this approach (approx. 79%).^17^ As only procedures with the same technique throughout the whole operation where enrolled in the present study, cases with conversions to stent retriever-based MT had to be excluded, leading to an artificially higher rate of successful ADAPT procedures among the included patients. We therefore listed the ADAPT results separated from the other techniques as there is only extremely limited comparability. For the other techniques, the conversion rates were very low (<1%). Besides the high conversion rate of ADAPT in the literature (21%–79%),^17,29–31^ this technique suffers another major drawback as the risk of distal embolization was shown to be significantly higher when using ADAPT as compared with Solumbra or BGC.^19^

**Figure 4.**
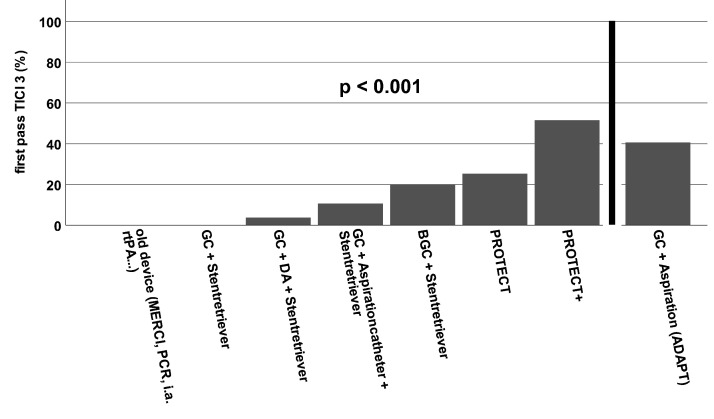
Rate of first-pass mTICI3 (complete) reperfusions that could be achieved by the different techniques analyzed here.

Since the very beginning of the stent retriever era in 2008 our institution implemented the “Solumbra” approach in endovascular stroke treatment. In the first years, distal access catheters were used that were replaced later by dedicated large bore aspiration catheters. This led to an improvement in important target parameters like mTICI3 reperfusion results (Figure 3), first-pass TICI 3 maneuvers (Figure 4), number of maneuvers, and procedure times.

As various studies in recent years have increasingly shown that the ultimate goal for the best possible clinical outcome for patients is a TICI3, i.e. complete, reperfusion,^32,33^ we also analyzed this factor.

**Figure 5.**
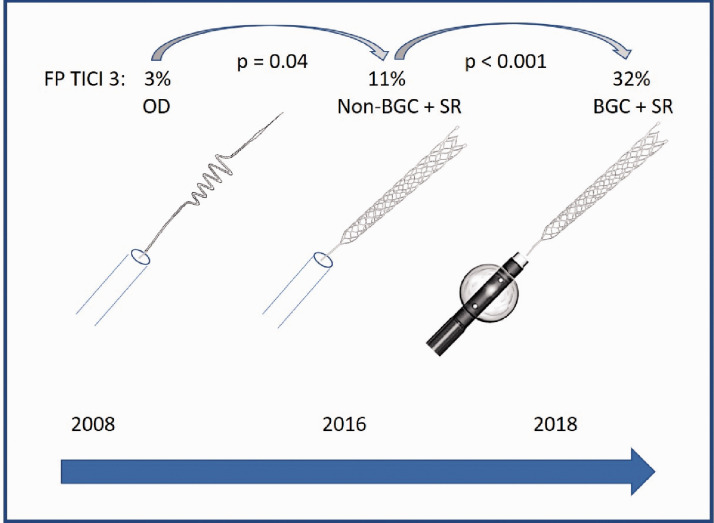
Increase of First-Pass (FP) TICI 3 rate interventions since 2008 comparing the “milestones” Old Device (OD), Non-Balloon Guide Catheter (Non-BGC) + stent retriever (SR), and Balloon Guide Catheter (BGC) + stent retriever.

Using old devices, complete reperfusion was achieved in only 12% of cases, using the latest technique – PROTECT^PLUS 24^- it was achieved in almost 76% of cases (Figure 3). In our opinion, probably the most valid parameter to define a "perfect" intervention is the combined parameter of the number of maneuvers and the final reperfusion result. The ultimate goal here is complete reperfusion in just one maneuver – first-pass TICI3. In the beginning, this was a relatively rare event that could first only be achieved with the use of a second-generation stent retriever (Table 1, Figure 5). With the additional use of BGCs, there was a further continuous increase, and the latest reperfusion technique using PROTECT^PLUS^ ^24^ achieved a first-pass TICI3 rate of 51.5% (Figure 4). Interestingly, we observed an overall improvement in technical parameters when BGCs were used instead of normal guiding catheters regardless of the exact technique, which is in line with the literature.^21,22^ Additionally the significant improvements from the era of “Old Device” to Non-BGC based interventions and even more impressively from non-BGC based interventions to interventions using BGC in combination with stent retrievers regarding the rates of first-pass TICI3 reperfusions (Figure 5) reflect the great advancements in thrombectomy techniques based on the available materials.

Our study is not without limitations. First, it is a retrospective analysis of a prospectively collected database with all its inherent restrictions. Second, the groups “GC + stent retriever” and “BGC + stent retriever” are rather small (n = 12 and n = 10, respectively). Therefore, the technical success rate of these two groups can only partially be considered realistic.

We believe that the continuous technical improvement of the MT must be attributed to the advancements in materials and respective techniques. Additionally, there might have been a certain learning effect within our department over the years that might also have positively influenced our technical results. Such learning effects cannot be reliably distinguished from procedural improvements. However, there has been considerable turnover of the neurointerventional staff, and we found no significant difference in the years of experience of the respective interventional team for each technique during the study period (Table 1). Therefore, probably the above mentioned “learning bias” is of minor importance.

By excluding the patients which switched to another rescue technique, we might have excluded the more difficult cases which inherits another potential bias. However, we do not see an adequate possibility to include the converted cases, since then, by nature, no assignment to an exact technique would be possible. In principle, this problem applies to all techniques, which in our eyes reduces this bias to an acceptable degree.

Unfortunately, no reliable, consecutive data on the long-term clinical course of our patients are available, as they have only been collected prospectively for the last few years in our institution. However, all studies published so far have clearly suggested that successful, complete, and first-pass reperfusion correlate with the clinical outcome. Thus, we assume that the continuous technical improvement also results in a direct clinical benefit for our patients.

## Conclusion

Even after the implementation of stent retrievers, the technical success of MT has further improved substantially over the past decade owing to improved materials and procedural innovations. At present, combination techniques including flow modulation have emerged to be the safest and most effective approach.
